# Early animal farming and zoonotic disease dynamics: modelling brucellosis transmission in Neolithic goat populations

**DOI:** 10.1098/rsos.160943

**Published:** 2017-02-15

**Authors:** Guillaume Fournié, Dirk U. Pfeiffer, Robin Bendrey

**Affiliations:** 1 Veterinary Epidemiology, Economics and Public Health group, Department of Production and Population Health, Royal Veterinary College, University of London, Hawkshead Lane, North Mymms, Hatfield AL9 7TA, UK; 2 School of Veterinary Medicine, City University of Hong Kong, Kowloon, Hong Kong; 3 Department of Archaeology, University of Reading, Whiteknights Box 226, Reading RG6 6AB, UK; 4 School of History, Classics and Archaeology, University of Edinburgh, William Robertson Wing, Old Medical School, Teviot Place, Edinburgh EH8 9AG, UK

**Keywords:** archaeology, epidemiology, mathematical modelling, animal domestication, one health, emerging disease

## Abstract

Zoonotic pathogens are frequently hypothesized as emerging with the origins of farming, but evidence of this is elusive in the archaeological records. To explore the potential impact of animal domestication on zoonotic disease dynamics and human infection risk, we developed a model simulating the transmission of *Brucella melitensis* within early domestic goat populations. The model was informed by archaeological data describing goat populations in Neolithic settlements in the Fertile Crescent, and used to assess the potential of these populations to sustain the circulation of *Brucella*. Results show that the pathogen could have been sustained even at low levels of transmission within these domestic goat populations. This resulted from the creation of dense populations and major changes in demographic characteristics. The selective harvesting of young male goats, likely aimed at improving the efficiency of food production, modified the age and sex structure of these populations, increasing the transmission potential of the pathogen within these populations. Probable interactions between Neolithic settlements would have further promoted pathogen maintenance. By fostering conditions suitable for allowing domestic goats to become reservoirs of *Brucella melitensis*, the early stages of agricultural development were likely to promote the exposure of humans to this pathogen.

## 1. Background

The shift from hunting and gathering wild food resources to the control and husbandry of domestic animals had fundamental and far-reaching repercussions for the evolution of infectious diseases in humans [1,2]. Through bringing animals together in larger, denser herds, in close association with human communities, a stable conduit for exposure of humans to infection in their animals was established [3,4]. Examination of the changing dynamics of human–animal relationships at the start of farming can not only advance understanding of the consequences of farming on human and animal health and wellbeing, but also contribute long-term perspectives to present and future concerns as animal management evolves to ensure sufficient and reliable food supply for the ever-growing global human population which in turn has resulted in changing environments.

However, while the origins of zoonoses as a consequence of the adoption of farming have been frequently hypothesized, there is little evidence in support of this supposition from archaeological records. Here, we discuss the origins of brucellosis as a zoonotic disease, a process that has been hypothesized as intensifying during the early period of animal domestication in the Near East [5,6]. *Brucella melitensis* is the main agent responsible for human brucellosis, today's commonest bacterial zoonosis in the world [7]. Humans become infected through ingestion of unpasteurized dairy products and the management of infected animals, primarily sheep and goats, the main reservoir of the bacteria [6]. A recent review of early evidence for brucellosis in human (*Homo sapiens*) skeletons identifies that the earliest probable cases reported come from the Bronze Age Near East [5], the region of domestication of goats and sheep, and also cattle and pigs, in multiple centres during the preceding Neolithic [8,9]. A further possible case derives from the early Neolithic Near East in association with evidence for early goat husbandry (an adult male skeleton (GD#22) from the site of Ganj Dareh exhibiting new woven bone on the anterior and lateral surfaces of a thoracic vertebral body and resorption of the superior anterior surface of a lumbar vertebral body removing a portion of the annular ring; both signs indicative of early brucellosis infection) [10]. A further possible case of brucellosis is also reported from a 2.4 to 2.8 Myr old hominin (*Australopithecus africanus*) skeleton [11]. While it indicates that humans may have been infected through contact with wildlife prior to animal domestication, the development of animal farming is likely to have further enhanced the risk of human infection by increasing (i) the prevalence of infection among in-contact animal populations and (ii) the frequency of contact between humans and infected animals through, for instance, the emergence of milk exploitation.

To explore the potential impact of the development of animal farming on brucellosis dynamics in domestic goats, and, therefore, on the risk of human infection, we consider the dynamics of *Brucella melitensis* infection in early domestic goat herds through a stochastic and age-structured mathematical model simulating its spread within village goat populations. The aim is to gain understanding of when in the evolution of goat husbandry conditions were reached for these animal populations to have the potential to sustain bacterial circulation within a settlement and to become a permanent reservoir for human infection.

**Figure 1.**
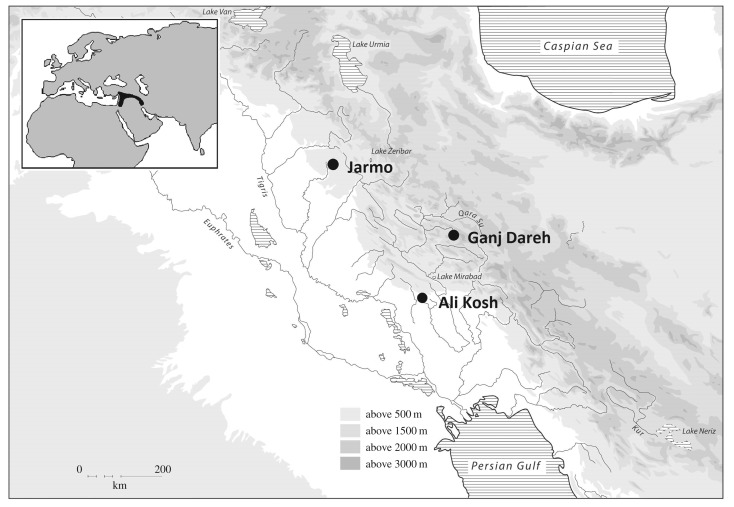
Location map showing Ganj Dareh, Ali Kosh and Jarmo in the eastern Fertile Crescent. Inset: region of early plant and animal domestications in the Near East known as the Fertile Crescent.

Current evidence indicates the emergence of goat husbandry in potentially multiple centres across the Near East during the late ninth/early eighth millennia BC [8,9]. Across the region, there is a range of evidence for increasing levels of management of goats at this time. For example, morphologically wild goats were transported to Cyprus, appearing as early as 8400 BC at Shillourokambos [12]. The early management of goats is reported at Nevalı Çori (*ca* 8200–8000 BC) in the upper Euphrates basin in the northern Fertile Crescent on the basis of size changes and demographic profiles [13]. Further east, early managed goats are also identified at the site of Ganj Dareh in the Zagros mountains of the eastern Fertile Crescent at *ca* 7900 BC, where the demographic profile indicates a population under human management that are morphologically unaltered from wild animals [14,15]. Archaeological sites from the Zagros region offer an ideal case-study for articulating a demographic model of early domestic goat populations to investigate the possible dynamics of brucellosis in the early stages of husbandry due to the well-dated and characterized sequence of site assemblages and the fact that for around a millennium goat was the only domestic food animal in the region [16]. The area has produced the earliest and most accurately dated demographic profile suggestive of a managed population at Ganj Dareh in the highland Zagros (figure 1) [14,15], where possible indicators of brucellosis were identified in a human skeleton [10]. The spread of goat husbandry can then be followed to nearby lowland zones, reaching Ali Kosh by *ca* 7500 BC, and Jarmo a few centuries later still where it co-occurs with domestic sheep [16] (figure 1). Examining these three populations—from Ganj Dareh, Ali Kosh and Jarmo—allows an assessment of the diversity in management strategies during earlier phases of animal husbandry [15] and the impact of these strategies on the potential maintenance of *Brucella melitensis* within domestic goat populations. With unpasteurized milk being a particularly key mode of transmission to humans [6], the origins of dairying are highlighted as an important innovation. Although the precise antiquity of dairying is still debated, zooarchaeological studies of herd profiles provide indirect evidence to suggest that milking may have begun in the Near East during the eight millennium BC [12,17]; whereas the earliest direct evidence comes from organic residues preserved in pottery from 7th millennium BC Anatolia [18].

## 2. Material and methods

The modelled goat population demographic profiles were defined using post-cranial remains found at the sites of Ganj Dareh, Ali Kosh and Jarmo as identified, recorded and reported by Zeder [16]. Although calculating demographic profiles from bone fusion provides less detail than from teeth eruption and attrition, importantly, it does allow calculation of sex-specific age profiles (not possible from dental data) due to sexual dimorphism in the goat post-cranial skeleton [14,16]. In the following, goats less than 1 year old are referred to as young, goats between 1 and 2 years old as yearlings, and goats of 2 years old or more as adults.

### 2.1. Population dynamics

Individual goats were the unit of analysis, and the model was run in discrete time with a daily time-step. New goats entered into the population through births. Births were seasonal, with the kidding season lasting *θ* days per year. While seasonal births meant that the size of the population varied over time, the average size of a goat population was stable from one year to the next. All goats born within the same season defined a cohort. Goats could leave the population at any time through harvesting or death due to other causes. The probability *δ_as_* of a goat dying between days *d* and *d* + 1 depended on its sex *s* and the age *a*, in years, of its cohort. Let *N_asd_* be the number of goats of sex *s* in a cohort of age *a* on day *d*. The number of goats dying on day *d* + 1 was simulated by a binomial process with *N_asd_* as the number of trials, and *δ_as_* as the probability of a success. When a cohort reached the maximum age Ω, it was removed from the population. The number of new born kids joining the population over a day of a given birth season was simulated by a Poisson process with *ωΠ*/*θ* as the average number of events. *ω* was the average litter size, i.e. the average number of kids per year and per female of kid bearing age, *θ* was the length, in days, of the kidding season and Π referred to the average number of females of kid bearing age (more than or equal to 2 years old) during a kidding season.

### 2.2. *Brucella melitensis* infection and transmission

Homogeneous mixing within the goat population of a village was assumed. Infected kids were assumed to be non-infectious, to recover from infection and be fully susceptible when reaching 1 year of age [19]. The infectious material excreted from the vaginal tract of infected goats following abortion or full-term parturition is generally considered to be the main source of infection for susceptible hosts [19]. While *Brucella* can also be shed in the semen, transmission is uncommon during natural mating [19,20]. Therefore, only female goats were considered to be potentially infectious, with transmission occurring through contacts with infectious material excreted following abortion or full-term parturition. It has been reported that infected goats may either be infectious for one abortion or parturition, or remain persistently infectious, with intermittent shedding [19]. Models published in the literature assumed either that infected hosts were infectious for only a couple of months [21], or remained infectious until their death [22]. In order to reflect this variability and uncertainty related to the course of infection of *Brucella melitensis* in goats, two scenarios were modelled. Under the lifelong infectiousness scenario, the goat population was divided into three mutually exclusive health states: Susceptible, Latent and Infectious. Infected goats entered into the latent state, and only females became infectious from their first abortion or full-term parturition since infection. Infectious females shed bacteria in fetal fluid and vaginal discharges for a period of ε days each year, following each abortion or full-term parturition. Under the transitory infectiousness scenario, the population was divided into four mutually exclusive health states: Susceptible, Latent, Infectious and Non-Infectious. Infectious goats only shed bacteria for a period of ε days following their first abortion or full-term parturition since infection. They then became non-infectious, and could not become susceptible or infectious again. The non-infectious health state included goats that recovered from *Brucella* infection and became immune, as well as goats that were permanently infected but did not shed the bacteria any longer.

*Brucella* transmission was assumed to be frequency-dependent, as this mode of transmission seems to be the most suitable for describing transmission in extensive production systems. Results assuming density-dependence transmission are also provided in the electronic supplementary material. The probability *p_ad_* of any susceptible goat in a cohort of age *a* becoming infected on a day *d* to *d* + 1 was expressed as follows:

$$\text{If}\;a=0,\;p_{ad}=0$$

and

$$\text{if}\;a\geq 1,\;p_{ad}=1-\exp\left(-\beta \frac{\sum_{l}I_{ld}^{S}}{\sum_{l,s}N_{lsd}}\right),$$

where $I_{ld}^{S}$ was the number of infectious (female) goats in a cohort of age *l* that shed bacteria on day *d*, i.e. within the ε-day period following abortion or parturition. *β* was the *per capita* number of effective contacts per unit of time (i.e. a contact resulting in infection if it involved an infectious goat). The number of goats in a cohort of age *a* becoming infected between day *d* and *d* + 1 was then simulated using a binomial process with the number of susceptible goats in that cohort as the number of events and *p_ad_* as the probability of a success.

In the metapopulation model, the probability *p_avd_* of any goat in a cohort of age *a*, in village *v*, becoming infected between day *d* and *d* + 1 was expressed as:

$$\text{If}\;a=0,\;p_{avd}=0$$

and

$$\text{if}\;a\geq 1,\;p_{avd}=1-\exp\left[-\beta \left((1-\alpha )\frac{\sum_{l}I_{lvd}^{S}}{\sum_{l,s}N_{lsvd}}+\frac{\alpha }{\left(n-1\right)}\sum_{i,i\neq v}\frac{\sum_{l}I_{lid}^{S}}{\sum_{l,s}N_{lsid}}\right)\right].$$

With *α* the proportion of contacts that a goat makes with goats from other villages, and *n* the number of villages. The first component of the exponent in this equation was the within-village infection process, and the second component was the between-village infection process.

### 2.3. Parameters

The demographic profile of goat populations was modelled using four daily probabilities of mortality: for young goats regardless of their sex, *δ_a_* _=_ _0_, for male and female yearlings, *δ_a_*_=1, *s*=*M*_ and *δ_a_*_=1,*s*=*F*_, and adults, *δ_a_*_≥2_. The model was first fitted to the survival probabilities assessed at each of the three archaeological sites (further details in the electronic supplementary material). This resulted in values of *δ_a_*_=0_ which were lower than expected [23] and which varied across sites. It may have been due to systematic errors—smaller and less dense remains of the youngest animals may have been preferentially destroyed by processes of taphonomic attrition—or variation in survival probabilities across sites, but was unlikely to reflect major differences in harvesting practices. Also, this first parametrization meant that the litter size (i.e. average number of kids by females at an age for bearing kids) required for a population size to remain stable from a year to the next differed between populations. As we were interested in assessing the impact of goat population management practices on disease dynamics, we adjusted all probabilities of mortality so that *δ_a_*_=0_ and the litter size remained constant across all sites (further details in the electronic supplementary material). A hypothetical, extensive, ‘modern’ goat population characterized by a lower ASR than for the three Neolithic populations, but a similar probability of mortality of young goats and average litter size was designed to assess the impact of increased male-biased harvesting on disease dynamics. For this ‘modern’ goat population, the sex ratio of 0.28 among goats more than 1 year old was comparable to the one reported in contemporary populations [24]. The range of explored values of *β* was selected so that, when applied to the ‘modern’ goat population, simulated seroprevalences (electronic supplementary material) covered the range of within-village seroprevalences reported by cross-sectional serological surveys conducted in the Middle East and Africa (5–35%) [21,25–27]. Other parameter values and estimation of goat population sizes at each site were assessed based on a review of the literature. Parameter values and details about their selection or calculations are provided in the electronic supplementary material.

### 2.4. Outcome

A simulation started by setting a random goat, on a random day, as infected. The probability of disease invasion was the proportion of simulations resulting in a substantial outbreak, defined as the infection of at least 50 goats. The disease was said to be endemic if there was at least one infected goat in the population after a period of 200 years. To calculate the probability of disease endemicity, only simulations that resulted in a substantial outbreak were taken into account. In the metapopulation model, the infection was seeded in a single village, and the disease was considered to be endemic if there was at least one infected goat among the *n* villages after 200 years. A thousand simulations were run for each parameter combination. The basic reproduction number *R*_0_ was estimated by calculating the dominant eigenvalue of the next-generation matrix **M** [28]. *R*_0_ informs about the potential for the bacteria to spread within a goat population: the disease may invade the population if *R*_0_ is higher than 1, while it will not if *R*_0_ is lower than 1. The entry *m_ij_* was interpreted as the expected number of newly infected goats of age *i* (in days) produced by one goat which became infected at age *j*, in an initially fully susceptible population. The calculation of *R*_0_ was checked numerically. As shown in figure 3*a*, the probability of disease invasion in the ‘modern’ goat population increased sharply when *R*_0_ became higher than one.

### 2.5. Sensitivity analysis

A global sensitivity analysis was conducted to assess the impact of variations in age- and sex-specific mortality probabilities on the minimum population size required for the probability of disease endemicity to be equal to or higher than an arbitrary value of 0.2. Parameter values were sampled using the Latin hypercube sampling scheme, and the partial rank correlation coefficients (PRCCs) were calculated, providing a measure of the influence of each parameter on the outcome. For each parameter, the range over which their value was varied was defined by [*r* − 0.1*r*, *r* + 0.1*r*], where *r* was the value of the corresponding parameter for Ganj Dareh demographic profile. PRCCs are provided in the electronic supplementary material.

All analyses were run using R v. 3.1.0. The package ‘sensitivity’ was used to conduct the sensitivity analysis.

## 3. Results and discussion

**Figure 2.**
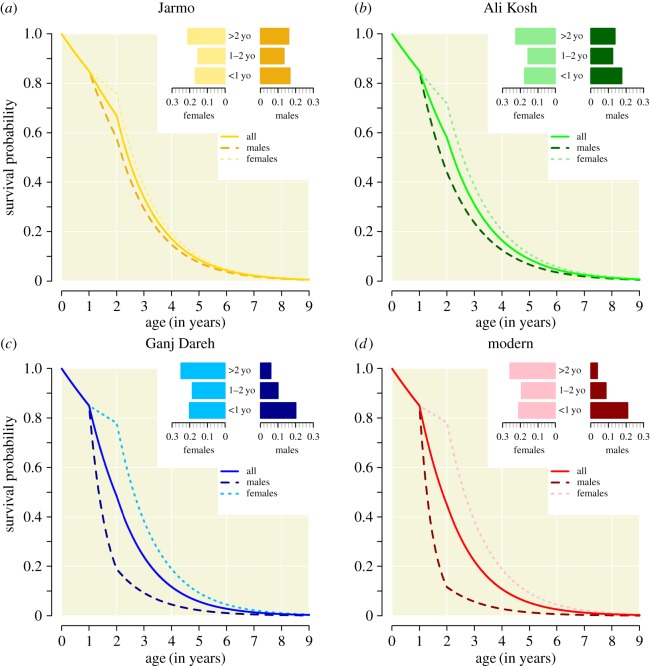
Village goat demographic profiles. Panels (*a*), (*b*) and (*c*) were inferred from post-cranial remains found in Jarmo, Ali Kosh and Ganj Dareh, respectively. Panel (*d*) refers to a hypothetical population reproducing features of ‘modern’ goat populations. Probabilities of survival as a function of age for the overall population (solid line), males (dashed line) and females (dotted line) are shown.

Demographic profiles in the three selected Neolithic sites were all characterized by a male-biased mortality of yearlings (figure 2). However, it was more pronounced in Ganj Dareh, resulting in a lower adult sex ratio (ASR = 0.24)—the ratio between the number of adult males and females—than at both Ali Kosh (ASR = 0.61) and Jarmo (ASR = 0.76). The higher mortality of males compared with females is likely to result from the selective harvesting of males. It is a common demographic structure for domestic livestock populations where only a few males are needed to ensure the reproductive continuity of the herd [29], and a feature of farming systems specialized in meat production (with most males slaughtered for consumption, while females are kept for reproduction), but may potentially also be associated with early dairy production strategies [30]. The low ASR in Ganj Dareh suggests the practice of such advanced levels of management aimed at increasing food production may have been applied at an early stage of the development of goat farming, although this pattern was by no means consistent across the Fertile Crescent [17]. A hypothetical, extensive, ‘modern’ goat population characterized by an even lower ASR (0.15) than Ganj Dareh was designed to further explore the impact of increased male-biased harvesting of yearlings on disease dynamics (figure 2).

**Figure 3.**
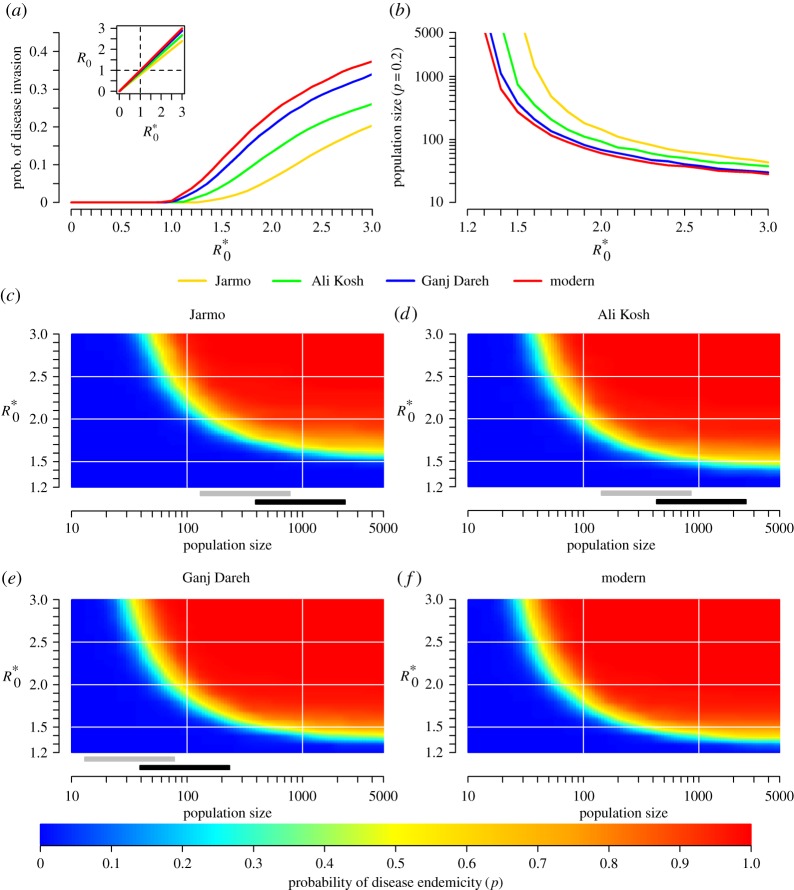
The probability of disease invasion and endemicity in a village goat population. Infectiousness was lifelong. $R_{0}^{*}$ is the value of the basic reproduction number *R*_0_ for the hypothetical ‘modern’ demographic profile. *R*_0_ informs about the potential for the bacteria to spread within the goat population: the disease may invade the population if *R*_0_ is higher than 1, while it will not if *R*_0_ is lower than 1. As population-specific *R*_0_ were linearly dependent (*a*), $R_{0}^{*}$ was chosen as a reference, and was reported on the *x*- or *y*-axes to allow comparisons between populations. (*a*) Probability of disease invasion as a function of $R_{0}^{*}$. (*b*) Minimum population size required for reaching a probability of disease endemicity *p* = 0.2 as a function of $R_{0}^{*}$. (*c*–*f*) Probability of disease endemicity as a function of population size and $R_{0}^{*}$. Grey and black bars show the ranges of estimated population sizes at each site assuming 100 and 300 people per hectare, respectively (electronic supplementary material).

As shown in figure 3 for the lifelong infectiousness scenario (and electronic supplementary material, figure S2, for the transitory infectiousness scenario), *Brucella melitensis* could invade (figure 3*a*) and be maintained for low levels of transmission in population sizes that were within the estimated ranges for the investigated Neolithic sites (grey and black bars in figure 3*c*–*e*; electronic supplementary material). These results suggest that conditions were present in these early domestic goat populations for the establishment of endemicity of the pathogen, which could thus have acted as a potential permanent reservoir for human infection.

The probability of mortality of male yearlings was the demographic parameter showing the highest level of variation across all four above-mentioned demographic profiles. It was also a highly influential parameter on the disease dynamics under both infectiousness scenarios (electronic supplementary material): for a given population size, the vulnerability to pathogen invasion and the probability of sustaining its circulation were higher in populations with high male-biased mortality, as in the Ganj Dareh and ‘modern’ profiles, compared with populations with low sex-biased mortality, as at Jarmo and Ali Kosh (lifelong infectiousness scenario: figure 3*b*; transitory infectiousness scenario: electronic supplementary material, figure S2*b*). In other words, the pathogen could be transmitted in such populations at disease transmission levels that did not allow it to be transmitted in comparable populations of the same size but for which harvesting was not biased towards males (further details about the impact of variations in the ASR on *Brucella* invasion and maintenance are presented in the electronic supplementary material). Likewise, the pathogen would circulate at a higher prevalence level in populations with high male-biased mortality. For a given population size and disease transmission level, the prevalence of infection was the highest in the ‘modern’ profile, and the lowest in Jarmo (electronic supplementary material, figure S5). However, the potential for the bacteria to become endemic in the Jarmo goat population may have been underestimated as sheep were also present [18] and likely to contribute to endemicity. Preferential harvesting of males increased the proportion of females in the population, and, therefore, their proportion among newly infected goats. As adult females are responsible for pathogen transmission, such a population structure would promote the transmission of *Brucella melitensis* (i.e. higher value of *R*_0_ for a given transmission rate).

**Figure 4.**
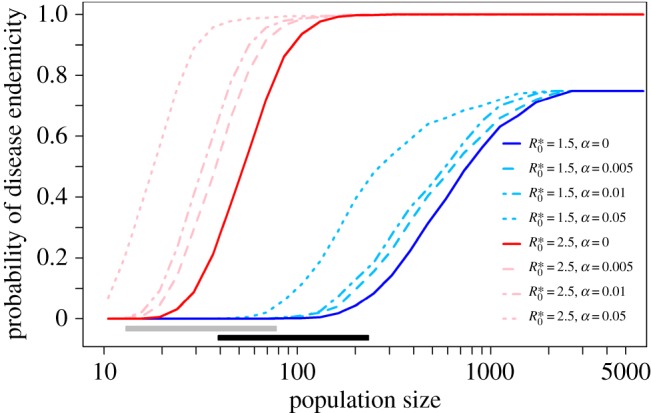
Impact of inter-population mixing on the potential for the disease to become endemic in a metapopulation of goats. Infectiousness was assumed to be life-long, and Ganj Dareh demographic profile was selected. *α* refers to the proportion of contacts that a goat made with goats from other villages. $R_{0}^{*}$ refers to the reference value of the basic reproduction number *R*_0_ for the hypothetical ‘modern’ demographic profile. Grey and black bars show the ranges of estimated goat population sizes at each site assuming 100 and 300 people per hectare, respectively (electronic supplementary material).

The potential for *Brucella melitensis* to become endemic was substantially increased if goats from different villages mixed together, even if the level of inter-village mixing was low (figure 4). A small metapopulation of 10 identical villages with demographic profiles similar to Ganj Dareh was simulated, with varying levels of contact between villages. In a metapopulation where goats made 0.5% of their contacts with goats from other villages (and, therefore, 99.5% of their contacts with goats from the same village), a probability of disease endemicity of, for instance, 0.2 was achieved for village populations 1.5 times smaller than villages in a metapopulation composed of isolated villages (figure 4). While disease extinction was more likely in these smaller populations, inter-village mixing could lead to the re-introduction of the pathogen in populations in which it faded out. This rescue effect meant that the pathogen could persist at the metapopulation level even if it could not persist at the population level [31]. Interactions between goats belonging to different villages were likely to occur during this period as Neolithic communities are known to have been linked by a broad range of regional interactions, including exchange networks amongst diverse other social relationships [32]. Such inter-village contacts may have resulted from the introduction of goats from one village to another, during seasonal transhumance practices [33], or the use of common pastures or water points. In the vicinity of Ganj Dareh, for example, several contemporaneous sites are situated within 2 or 3 h walking distance [34]. Moreover, contacts between human settlements may have further promoted the spread of certain population management practices, and especially the selective harvesting of young males, among these settlements.

The study has several limitations due to the nature of the data informing the model. Assumptions about the routes and modes of transmission of *Brucella* in Neolithic goats were based on our current understanding of the epidemiology of the disease. Infection of wild goat species by *Brucella* is common, and can reach high levels of prevalence [6,35]. As present-day domestic goats derive from Neolithic populations, we assumed that the main features of *Brucella* pathology, and especially the restriction of *Brucella* transmission to females, remained the same. While the explored values of *β* were selected to allow the amplification of the pathogen in the ‘modern’ goat population, it is unknown whether infectious contact rates in the Neolithic period were comparable to those observed nowadays. Further archaeological investigations and genetic analyses of ancient *Brucella* DNA would help in assessing the validity of these assumptions. Remains of goats that were not slaughtered and consumed, but died for other reasons (e.g. diseases), might have been discarded at a distance from the settlement, and smaller and less dense remains of the youngest animals may have been preferentially destroyed by processes of taphonomic attrition. This may have led to systematic errors in the estimation of survival probabilities. For comparison purposes, a hypothetical modern demographic profile was developed. Certain features, such as average litter size [36,37] and age at slaughter may vary between breeds and husbandry practices, and these variations were not captured here. Our main aim was to represent the impact of the sex-biased harvesting of goats observed in modern extensive goat flocks, and the resulting ASR, in order to allow a comparison with the different observed Neolithic demographic goat population profiles. We estimated possible sizes of goat populations at Ganj Dareh, Ali Kosh and Jarmo. Such calculations are a challenge for archaeological research given the nature of the evidence [38], but was attempted here to add perspective to the discussion of the relationship between population size and disease endemicity. The wide range of parameter values we used aimed to capture the uncertainty associated with these estimates, with the population size estimates spanning several orders of magnitude (electronic supplementary material). We assumed that managed goat population dynamics was independent from wild goat populations. However, early farmers might have regularly recruited wild animals and introduced them into their flocks. Such a practice could have resulted in multiple introductions of the pathogen into managed flocks, and could have, therefore, further promoted the likelihood of the pathogen being maintained in village goat populations.

In conclusion, the increase in livestock densities may not be the only feature resulting from the early development of farming that promoted disease invasion and maintenance. The alteration of goat population demographic profiles, probably associated with management decisions to increase productivity of herds, and likely interactions between settlements further increased the potential for these populations to spread and maintain infection. Through these changes in goat population dynamics and contact patterns, conditions promoting the exposure of humans to a zoonotic pathogen emerged at an early stage of farming development. In the earliest period of caprine husbandry across the Near East a diversity of management strategies were practiced, as communities experimented with differing herd profiles, with only a minority of Early Neolithic sites demonstrating pronounced young male kill-off [15]. This situation changes from the mid-7th millennium BC, after which the majority of sites produce clear evidence for young male kill-off of domestic caprines at the same time as a new emphasis on intensive and large-scale mixed sheep and goat pastoralism emerges [15]. Thus, zoonotic brucellosis had the potential for emergence in some geographical areas of the Early Neolithic, but may not have become widespread until the relevant management strategies were in wider use. Transmission to human communities would have been further enabled by the development of dairying practices, although whether or not milking was practiced in the Zagros Neolithic sites is currently inconclusive from the herd demographics.

Understanding of the interrelationship between disease dynamics and population characteristics within this broader regional narrative will allow future osteological and genetic research to focus on those areas most likely to produce direct evidence for the emergence of livestock-related zoonotic disease. To date, the study of the role of animal domestication in the emergence of brucellosis has been limited by what is identifiable from the archaeological records. Palaeopathological studies of human and animal remains can often indicate only non-specific infections, as identifying the causative agent based on structural changes within bone alone is problematic given the lack of specificity of these changes for the various pathogens and also only a proportion of individuals infected by an infectious organism might show evidence of skeletal changes [39,40]. Furthermore, the taphonomic histories of most animal bone assemblages (butchered, fragmented and cooked) act against the identification of diseases [41]. Analyses of ancient DNA have increasing potential for the identification of infectious agents, although they have been challenged to date by detrimental environmental conditions in the Near East to DNA survival in archaeological bones [42,43]. They are, however, beginning to contribute to the identification of brucellosis for later archaeological periods [44], and recent advances represent significant potential for the recovery of pathogen DNA from the ancient Near East [45]. Future directions should focus on analyses to investigate the presence and distribution of brucellosis in the archaeological populations, particularly through refinement and application of palaeopathological criteria for identifying brucellosis in goat skeletons, and application of ancient DNA analytical protocols. Such future analyses are needed to test the model results and validate conclusions. For example, we would expect to see higher prevalence rates at sites with population demographics promoting infection. Future work should also further refine and validate the parameters employed in the model, for example, data on timings and changes in birth seasonality can be generated from stable isotope analysis of archaeological teeth [46] and extend the simulations to later periods where we witness the development of more intensive husbandry systems [15] or the increase in occurrence of reported cases of brucellosis in humans [5,6]. Stable isotope analyses on archaeological remains could also provide information on the spatial mobility of goats, and, therefore, on the likelihood of interactions between goat flocks belonging to distinct settlements.

These findings further support the view that the transition from food collection to production during the Neolithic transition while allowing for larger human population sizes resulted in significant adverse effects on human health and wellbeing [2,47]. It further demonstrates the importance of recognizing the complexity of eco-social systems, where it is often very difficult to obtain a holistic impression of the different types of impacts that a particular change in the system has. In this case, early farmers discovered that they could improve the efficiency of food production while maintaining herd reproductive continuity by selectively culling young male goats, a cause–effect relationship that must have been clear to them. But they were unlikely to have realized that this led to increased risk of human brucellosis, due to the cause–effect relationship not being directly observable. Arguably, even if they had recognized the link, the perceived benefits of more effective food production and associated outputs may still have resulted in choosing the same goat herd production management approach.

## Supplementary Material

Supplementary material – Parameters and sensitivity analysis. In this supplementary material we provide further information about the choice and calculation of parameter values, and we describe the results of the sensitivity analysis.

## Data accessibility

This study uses data from the literature.
