# Supplementary material for: Early animal farming and zoonotic disease dynamics: modelling brucellosis transmission in Neolithic goat populations
Source: R Soc Open Sci. 2017 Feb 15;4(2):160943. doi: 10.1098/rsos.160943 (PMC5367282; doi:10.1098/rsos.160943)
Supplement: Supplementary material – Parameters and sensitivity analysis. In this supplementary material we provide further information about the choice and calculation of parameter values, and we describe the results of the sensitivity analysis. [file rsos160943supp1.pdf]

Early animal farming and zoonotic disease dynamics: modelling brucellosis transmission in Neolithic goat populations

Guillaume Fournié, Dirk U Pfeiffer, Robin Bendrey

Supplementary material – Parameters and sensitivity analysis

In this supporting information we provide further information about the choice and calculation of parameter values, and we describe the results of the sensitivity analysis.

Parameters. Details about the choice of parameter values are provided below. Parameter values are provided in table S1.

Maximum age of a goat: The archaeological postcranial remains suggested that goats could survive beyond the four year limit of the long bone fusion ageing method [1]. Nowadays, female goats may remain fertile for at least 10 years. However, in most husbandry systems, females are only retained to eight years old [2]. Here, we assumed that goats could not get older than nine years old. When a goat reached that age, it then left the population.

Age at first kidding: Nowadays, farmers often breed goats in their first year of life. However, breeding seems to occur later in extensive systems. Damascus, Mamber and Negev breeds which are commonly raised in the Near East are generally reported to give birth from 24 months onwards [3, 4]. Likewise, age at first kidding for wild goats (*Capra aegagrus*) was reported to be two years [5].

Length of the birth season: The birth season of Damascus breed, Negev breed and wild goats (*Capra aegagrus*) was generally reported to last from a couple of weeks to three months [4, 5].

Litter size: It was the average number of kids per year and per female of kid bearing age. Litter sizes for contemporary domestic goat breeds raised in the Near East region (e.g.

Damascus, Maltese, Mamber, Negev, Saanen) were reported to lie between 1.3 and 1.8 [3]. Twins were reported to be common in wild goats (*Capra aegagrus*) [5].

Length of the bacteria shedding period: Infected goats were reported to shed bacteria from the vagina for 2 to 3 months following abortion or full-term parturition [6]. They may also shed the bacteria in milk for extended periods during the lactation [6].

Probability of mortality of a goat of sex s in age category a: These age- and sex-dependent probabilities were assessed through two successive steps. For each archaeological site, the daily probabilities of death were first estimated by maximising a Poisson likelihood function:

$$L = \prod_s \prod_i \frac{e^{-\upsilon_{is}} {\upsilon_{is}}^{\Upsilon_{is}}}{\Upsilon_{is}!}$$

Υ_{is} was the number of goats of sex *s* having survived at age *i* in a cohort of *N* goats, based on survival probabilities derived from archaeological data and reported in table S2. *N* was arbitrarily fixed to 1000. υ_{is} was the modelled number of goats of sex *s* having survived at age *i*:

$$\begin{cases} i \le 365 \ days, \ \upsilon_{is} = N\left(1-\delta_{a=0}\right)^{i} \\ 365 < i \le 730 \ days, \ \upsilon_{is} = \left(1-\delta_{a=0}\right)^{365}\left(1-\delta_{a=1,s}\right)^{i-365} \\ i > 730 \ days, \ \upsilon_{is} = \left(1-\delta_{a=0}\right)^{365}\left(1-\delta_{a=1,s}\right)^{365}\left(1-\delta_{a\ge2}\right)^{i-730} \end{cases}$$

Results are shown in figure S1. In a second step, we aimed to keep the adult sex ratio (ASR) and the ratio $\delta_{a\ge2} \big/ \left(\delta_{a=1,s=F} + \delta_{a=1,s=M}\right)$ equal to those obtained with the abovementioned parameterisation, for each site. The following algorithm was conducted. The superscript * denoted the parameters obtained through the first parameterisation. First, new values of $\delta_{a=1,s=F}$ and $\delta_{a=1,s=M}$ were randomly drawn, such as $\delta_{a=1,s=M} > \delta_{a=1,s=F}$. Secondly, $\delta_{a\ge2} = \delta^*_{a\ge2}\left(\delta_{a=1,s=F} + \delta_{a=1,s=M}\right) \big/ \left(\delta^*_{a=1,s=F} + \delta^*_{a=1,s=M}\right)$, and $\delta_{a=0}$ was computed so that the probability that a newborn reached one year-old was equal to a value of 0.85 [4]. While this

value might have been too high, it would not have impacted on our comparison of the disease dynamics in the simulated goat populations, since it was the same across all sites and infected young goats were non-infectious and became susceptible again when reaching 1 year of age [6]. Finally, the litter size required for the population size to remain stable over the years and the ASR corresponding to the new parameter values were computed. This algorithm was repeated 1,000,000 times. The parameter values associated with a litter size and ASR being the closest to their targeted values were selected.

Neolithic village goat population size estimates: Goat population sizes were estimated as follows:

$$[goat\ population\ size] = \frac{[area\ size][human\ density]}{[household\ size]}[goats / household]$$

Ganj Dareh is a small settlement in the Zagros highlands (c. 1400m asl) covering about 0.13 hectare [7]. Ali Kosh is a larger site in the southern Zagros lowlands (c. 150m asl) covering 1.43 hectares [8]. Jarmo is situated in the western Zagros piedmont (c. 800m asl) covering about 1.3 hectares. Jarmo is thought to be a permanent, year-round settlement, home to an estimated 150 or more people [9]; whereas whether Ganj Dareh and Ali Kosh were year-round or just seasonal settlements is unclear and still debated.

Estimates vary considerably on the human population density of ancient villages. Here, two estimates are calculated to represent the potential range in population size. These are based on a lower density of 100 persons per hectare and an upper one of 300 hectares per hectare (table S3). There are Near Eastern ethnoarchaeologically derived estimates for both values. Köhler-Rollefson and Rollefson [10] calculated estimates for goat population sizes at the Neolithic site of Ain Ghazal, Jordan, based on a series of assumptions about the number of goats that would be consumed by every family group each year. They assume family group size as six people, which is also used here (table S3). It must be emphasized that published

estimates also vary for family size, mostly ranging between four and eight people per household [11]; and also that there is debate over the extent to which nuclear families operated as socioeconomic units in Near Eastern Early Neolithic communities.

Based on data in Dahl and Hjort [12], Köhler-Rollefson and Rollefson [10] assumed a ~30% annual off-take of animals from the flock (that for every goat consumed two further individuals of breeding stock would have been kept). They proposed three levels of consumption: low (each family has six goats, of which two juveniles are available as meat for each family each year), medium (each family has 18 goats, of which six juveniles are consumed each year) and high (each family has 36 goats, of which 12 juveniles are consumed each year). The same estimations are applied here. These calculations produce estimated total ranges for goat populations of 13-234 at Ganj Dareh, 143-2574 at Ali Kosh, and 130-2340 at Jarmo (table S3). It must be remembered that goat is the only domestic food animal that was husbanded at both Ganj Dareh and Ali Kosh, whereas sheep and possibly pig were also husbanded at Prepottery Neolithic Jarmo [1]. Hunted wild animals also provided a small proportion of meat to the diet at all sites.

Although the maximum probable size of a domestic animal flock is linked to the carrying capacity of the territory, it is possible to make some estimates of likely flock sizes based upon settlement sizes as inferred from excavations, estimations of human population densities for those sizes of settlements, and the likely number of livestock expected to be kept by those estimated human populations sizes. Opinions differ significantly on the variables and validity of such calculations. The sequences of inferences and estimations present an indication of possible human and goat numbers only, but do allow comparisons and contextualised discussion of the populations simulated in this paper. Kramer's research [13] on a small-scale rural settlement in the highland Iranian Zagros identified an average flock size of 44 sheep and goats, over 55 households. Spread over all 67 households of the settlement, including

those who did not have any animals, returns an average of 36 sheep and goats per household, or 5.7 animals per villager. These values from Kramer's study indicate that the estimates from the higher goat:human ratios are not unrealistic for the Neolithic settlements.

Sensitivity analysis. The influence of variations in age- and sex-dependent daily probabilities of mortality on the minimum population size required for the probability of disease endemicity to be higher than $p = 0.2$ was assessed. The ranges over which parameter values were varied were defined by reducing and increasing the baseline parameter values by 10%. Ganj Dareh estimates were chosen as references (table S1). Values of β corresponding to values of R_0^* equal to 1.5 and 2.5 under both transitory and lifelong infectiousness scenarios were used. Partial rank correlation coefficients are shown in table S4.

Under both infectiousness scenarios, the probability of mortality of male yearlings and of adults were the most influential parameters. As the probability of mortality of male yearlings increased, and the probability of mortality of adults decreased, the population size required to maintain *Brucella* was reduced. In other words, the potential of the population to maintain *Brucella* increased. Under the transitory infectiousness scenario, the influence of the probability of mortality of adults on the outcome of disease invasion decreased as β increased. If the infectiousness is transitory and the life expectancy of adult goats rises, then the likelihood that infected goats become infectious and shed the bacteria for the duration of their infectious period increases. This would also increase the likelihood that infected goats live long enough to become non-infectious. Yet, as transmission increases, goats become infected at a younger age, and, if their life expectancy is high, then the proportion of non-infectious goats in the population would rise, reducing the potential of the population to sustain *Brucella* circulation. In contrast, under the lifelong infectiousness scenario, infected

goats remained infectious until their death, and a longer life expectancy would increase the duration of *Brucella* shedding.

The difference in the potentials of Ganj Dareh and Ali Kosh demographic profiles to sustain *Brucella* transmission seemed more pronounced in the transitory (figure S2) than the lifelong infectiousness scenario (figure 3). It can be explained by the higher influence of the probabilities of mortality of male and female yearlings, and the lower influence of the probability of adult mortality under the transitory than the lifelong infectiousness scenario.

Results obtained assuming density-dependent transmission are similar to those obtained assuming frequency-dependent transmission (figure S3, figure S4).

Figure S5 shows the average prevalence of infection at the endemic equilibrium as a function of R_0^* for the 4 demographic profiles. The prevalence of infection is the highest in the "modern" profile, and the lowest in Jarmo.

Impact of variations in the adult sex ratio on the invasion and maintenance of Brucella.

We further explored the impact of variations in the adult sex ratio (ASR) on the potential of a goat population to amplify and maintain the circulation of *Brucella*. ASR was modified by altering the daily probability of mortality of male yearlings. Other parameters were fixed. Litter size and daily probability of mortality of young were as specified in table S1. Two sets of parameters were chosen for the probability of mortality of female yearlings and adults, covering the ranges of parameter values estimated for the 4 demographic profiles (table S1): (1) $\delta_{a=1,s=F} = 4.6*10^{-4}$, $\delta_{a=2} = 17.1*10^{-4}$ and (2) $\delta_{a=1,s=F} = 2.1*10^{-4}$, $\delta_{a=2} = 19.6*10^{-4}$. As the two sets of parameters resulted in the same conclusions, only results generated with the first set of parameter are presented here.

As shown on figure S6, R_0 increased as the ASR decreased – in other words, R_0 increased with the preferential harvesting of males. Compared to a goat population characterised by an

ASR of 1 (i.e. unbiased harvesting of males and females), R_0 increased by 42% if the ASR was reduced to 0.11 (i.e. 90% of adult goats were females).

The influence of variations in ASR, population size and β on the probability of *Brucella* endemicity was assessed through a global sensitivity analysis. The ranges over which parameter values varied were: 0.11 to 1 for ASR (i.e. proportion of females among adults ranging from 50% to 90%); values of β corresponded to values of R_0^* ranging from 1.2 to 3; the population size ranged from 50 to 2500, covering the range of plausible values estimated for the three archeological sites. While the ASR had a substantial impact on the probability of disease endemicity (table S5), it was less influential than β and the population size, under both infectiousness scenarios (table S5). Figure S7 shows the probability of disease endemicity in a goat population as a function of the ASR and population size. For low levels of *Brucella* transmission, a small variation in the ASR could drastically impact the potential of a goat population to maintain the bacteria circulation. Reduction of the ASR first created conditions for bacteria amplification (i.e. R_0>1). For instance, under the transitory infectiousness scenario and for a value of β corresponding to $R_0^* = 1.5$, the bacteria circulation could only be maintained for low values of ASR, even if the goat population was large. If the value of ASR allowed R_0>1 (eg ASR=0.5), a two-fold reduction of ASR, from 0.5 to 0.25, resulted in a five-fold reduction of the population size required for reaching a probability of *Brucella* endemicity of 0.2. As the level of transmission increased, the impact of the ASR was reduced in comparison to the impact of the population size. Decreasing the ASR from 0.5 to 0.25 only meant that the minimal population size required for reaching a probability of endemicity of 0.2 was divided by 1.5 for $R_0^* = 2$, and by 1.2 for $R_0^* = 2.5$.

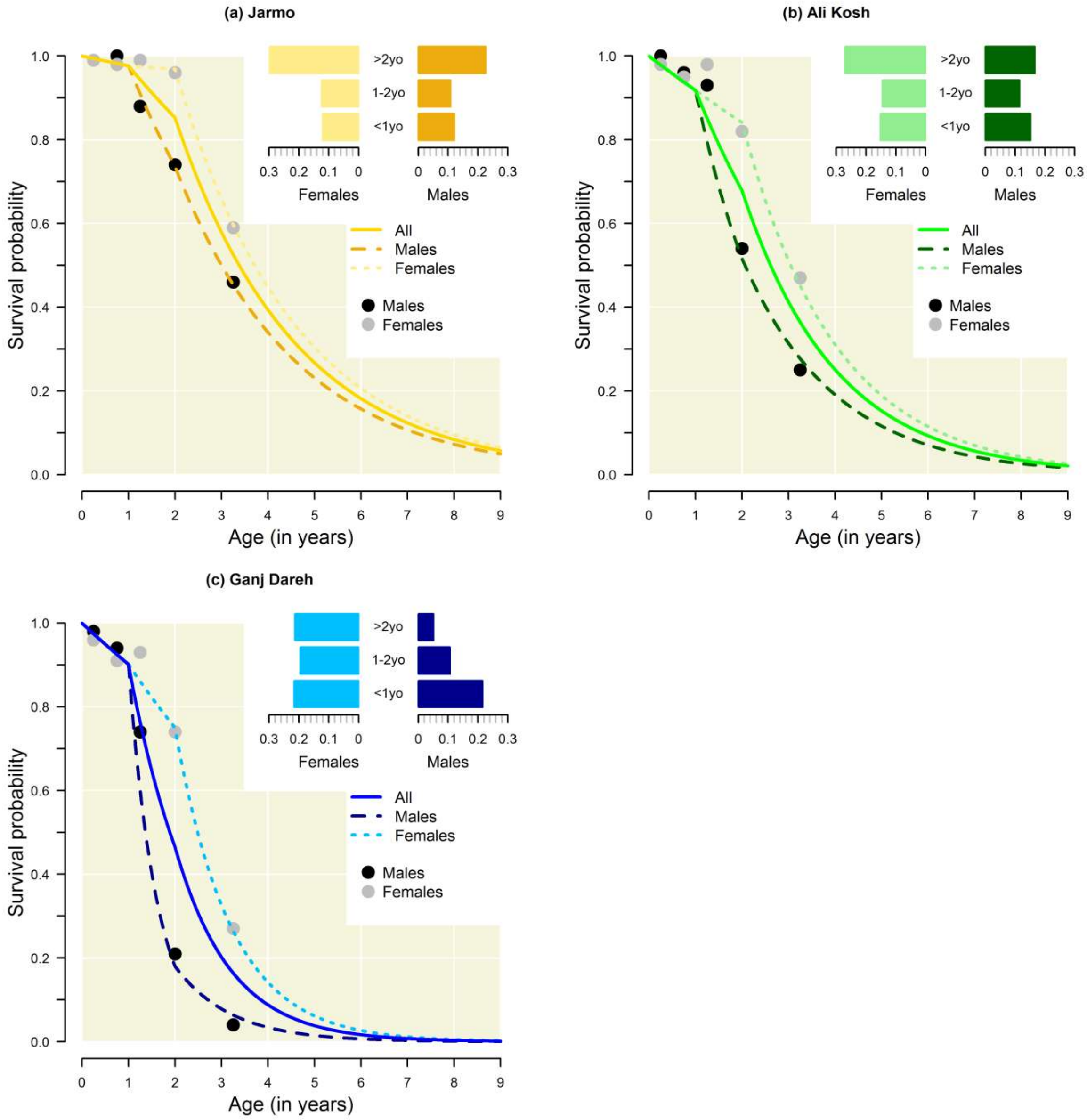

Figure S1. Non-adjusted village goat demographic profiles. (A), (B) and (C) were inferred from postcranial remains found in Jarmo, Ali Kosh and Ganj Dareh, respectively. The probabilities of survival estimated from postcranial remains for males (black dots) and females (grey dots), and the modelled probabilities of survival as a function of age for the overall population (solid line), males (dashed line) and females (dotted line) are shown.

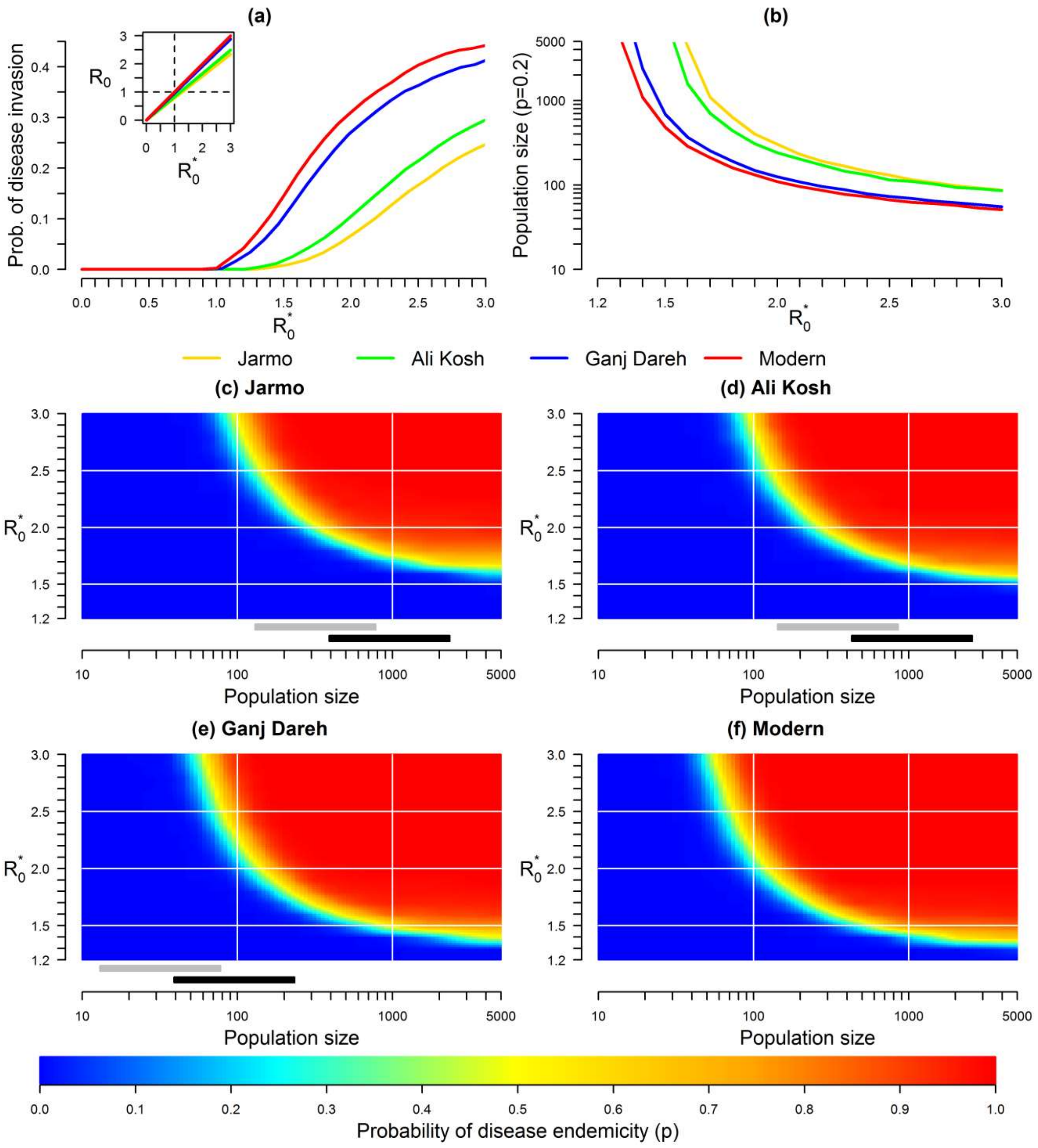

Figure S2. The probability of disease invasion and endemicity in a village goat population under the transitory infectiousness scenario. The legend is similar to figure 3.

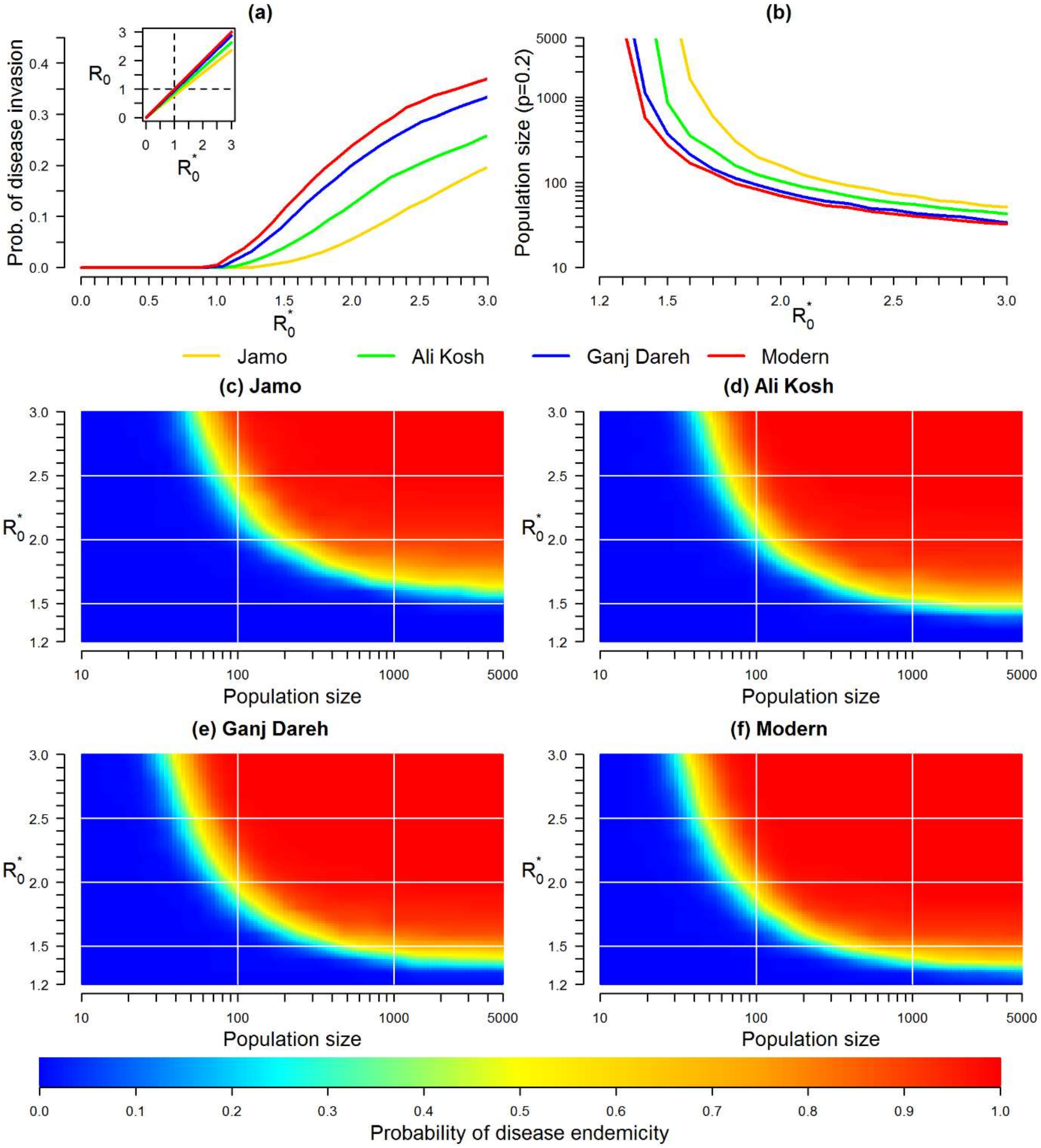

Figure S3. The probability of disease invasion and endemicity in a village goat population under the density-dependence mode of transmission and the lifelong infectiousness scenario. The legend is similar to figure 3.

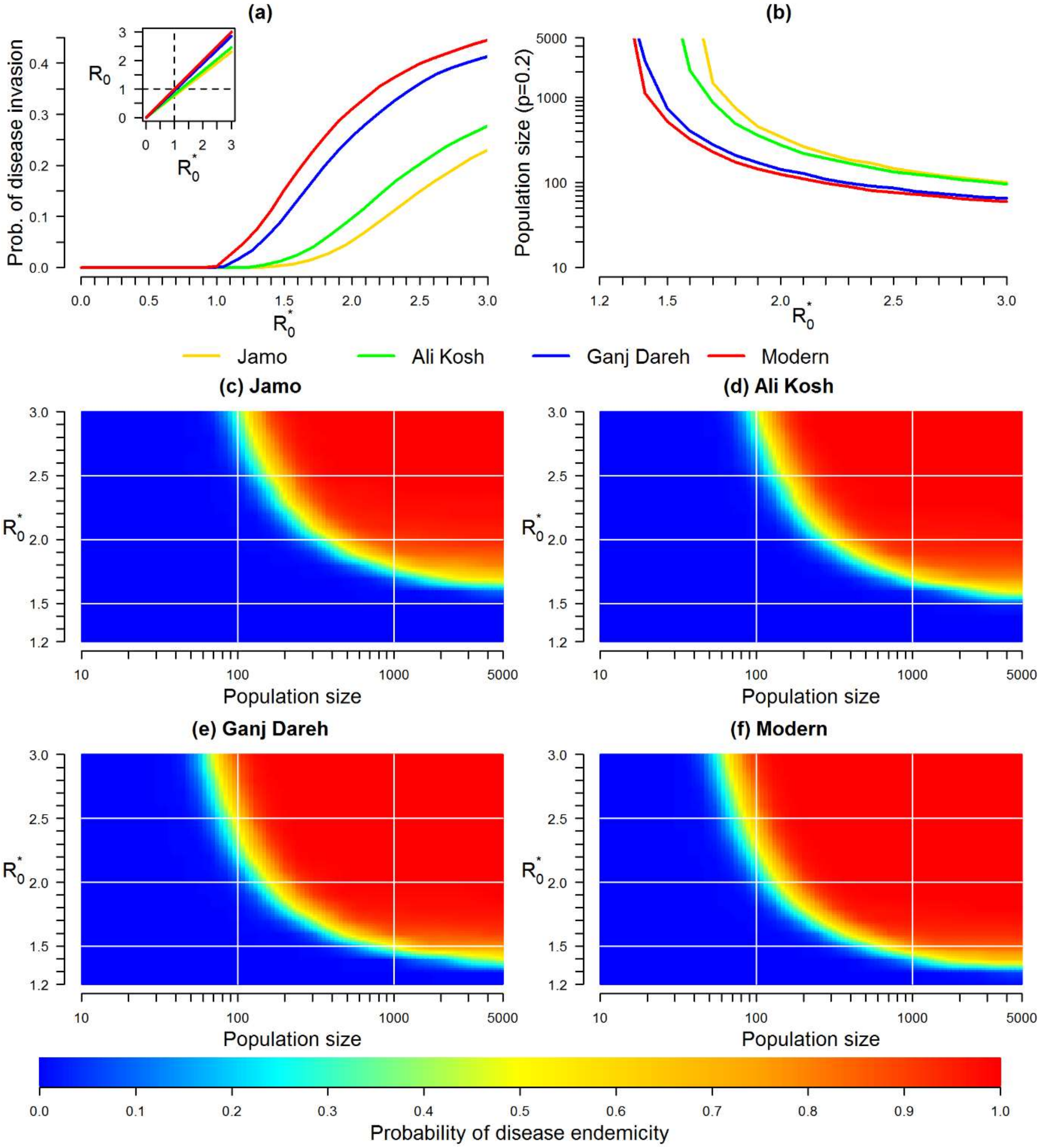

Figure S4. The probability of disease invasion and endemicity in a village goat population under the density-dependence mode of transmission and the transitory infectiousness scenario. The legend is similar to figure 3.

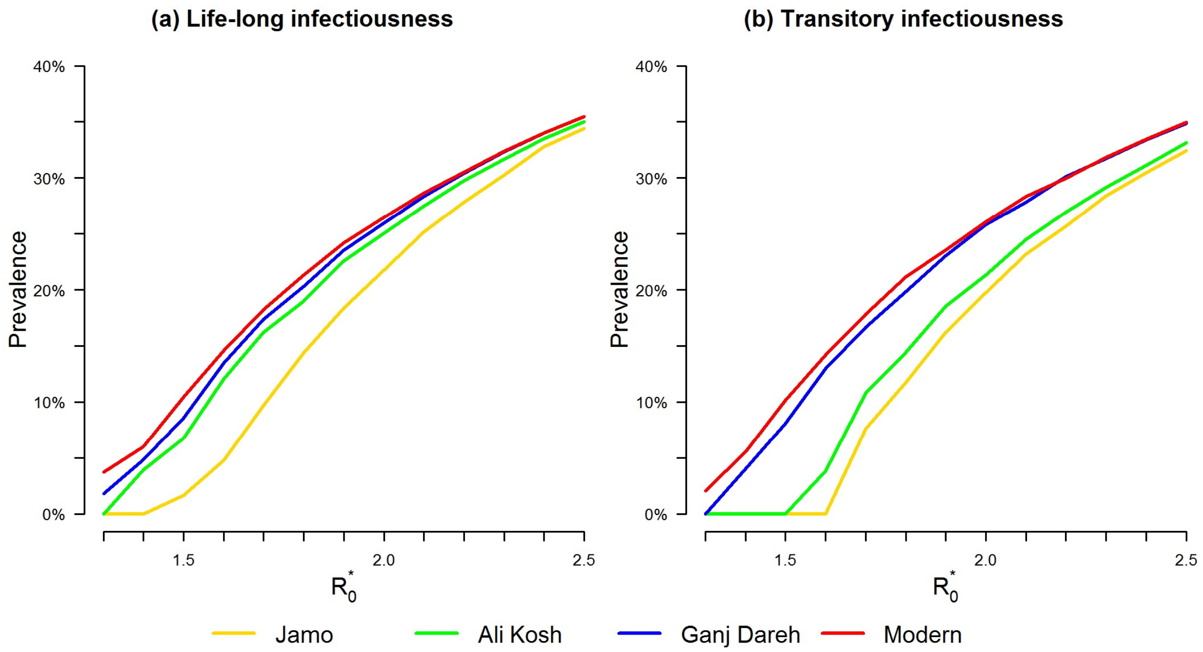

Figure S5. Average prevalence of *Brucella melitensis* infection in a goat population at the endemic equilibrium. There are 1000 goats in each population. Only simulations for which brucellosis reached the endemic equilibrium were accounted for in the calculation of the average prevalence. The prevalence was the ratio between the number of latent, infectious and non-infectious goats and the total number of goats.

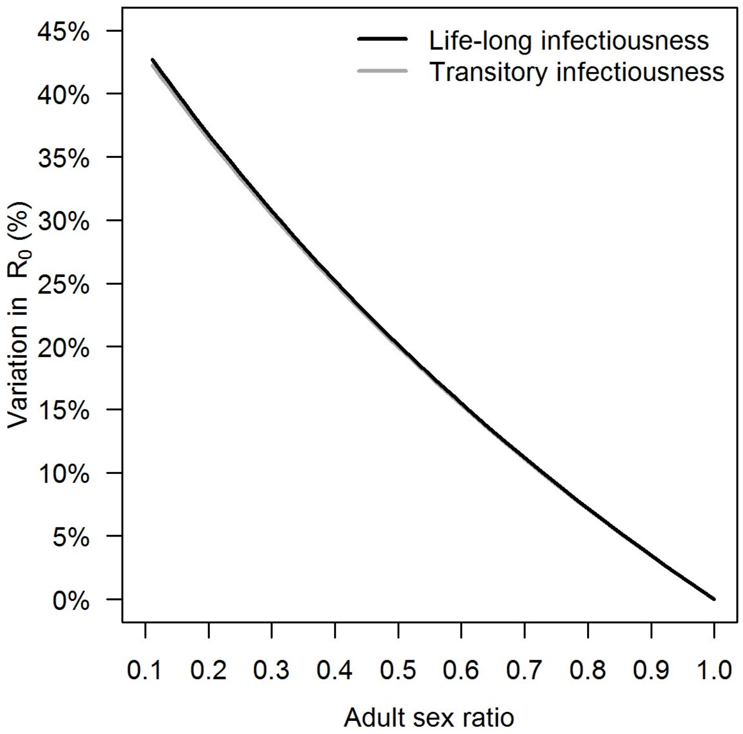

Figure S6: Variations in R_0 caused by changes in the adult sex ratio. The adult sex ratio (ASR) is the ratio between the proportion of adult males and females in the population. Values of R_0 are compared to the value of R_0 for ASR=1. Results are shown for the life-long and transitory infectiousness scenarios.

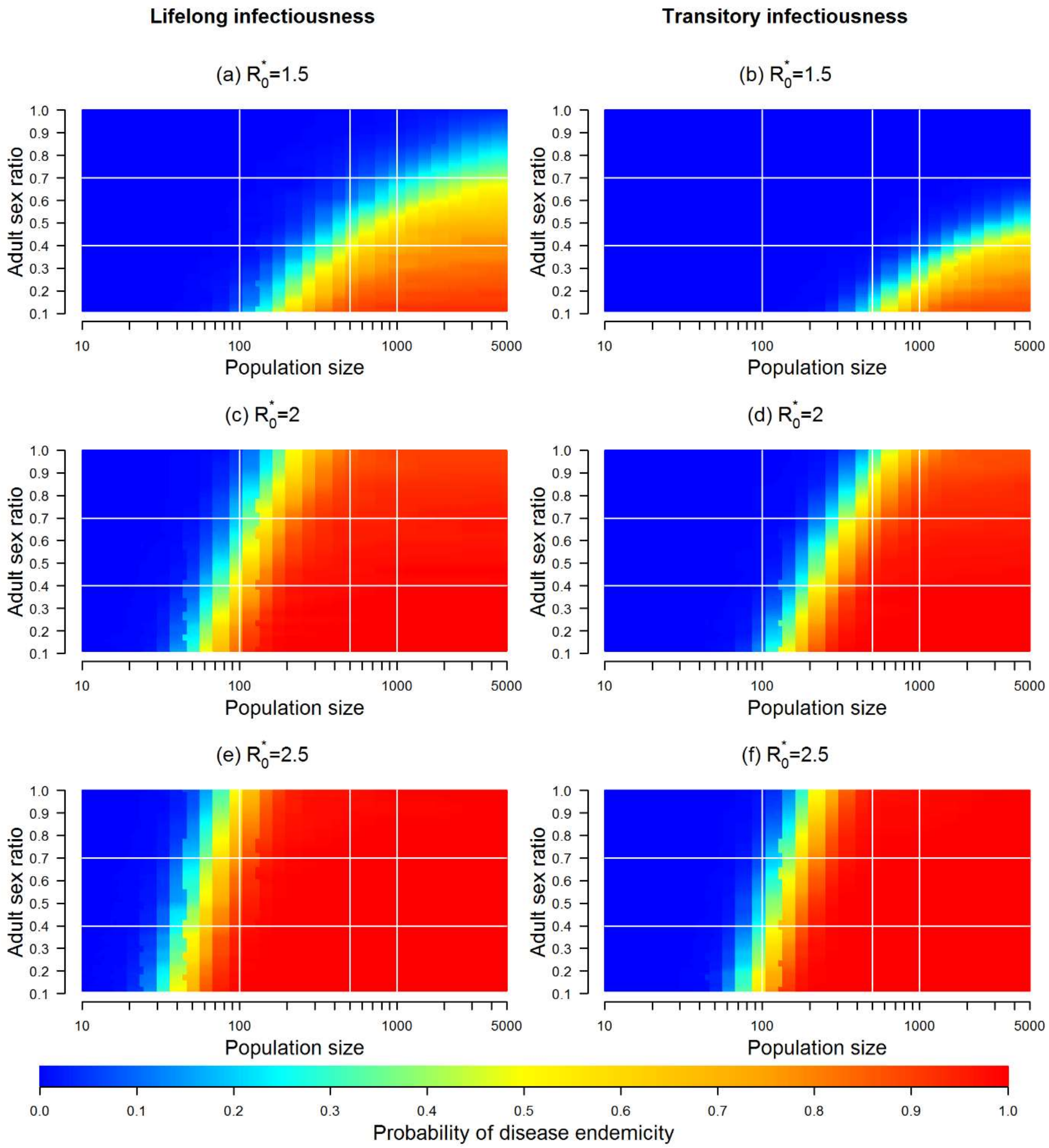

Figure S7: The probability of disease endemicity in a village goat population as a function of the adult sex ratio and the population size. The adult sex ratio (ASR) is the ratio between the proportion of adult males and females in the population.

Table S1. Parameter values. Adj: Adjusted. J: Jarmo; A: Ali Kosh; G: Ganj Dareh.

Parameters	Description	Value (unit)	Ref
K	Age at first kidding	2 (years)	(*21, 23, 24*)
Θ	Length of kidding season	1 (month)	(*21, 24*)
Ω	Maximum age of a goat	9 (years)	
ω	Average litter size	1.3	(*23, 24*)
ε	Infectious period following abortion or parturition	90 (days)	(*18*)
$\delta_{a=0}$	Daily probability of death, youngs (x 10^{-4})	J:4.5<br>A:4.5<br>G:4.5<br>M:4.5	
$\delta_{a=1,s=M}$	Daily probability of death, male yearlings (x10^{-4})	J:10.5<br>A:18<br>G:41.1<br>M:54.3	
$\delta_{a=1,s=F}$	Daily probability of death, female yearlings (x10^{-4})	J:3<br>A:4.6<br>G:2.4<br>M:2.3	
$\delta_{a\geq 2}$	Daily probability of death, adults (x10^{-4})	J:18.6<br>A:17.2<br>G:19.4<br>M:19.5	

Table S2. Goat survival probabilities as a function of age, sex and location estimated based on long bone fusions, following [1]. In [1], the survival probability of males of age category D in Jarmo was 0.88 in Table 1, but was about 0.74 in Fig. 6. Here, ages were the average of the age categories reported in [1]. Survival probabilities of 15 months-old goats, estimated based on the fusion of phalanges, were ignored as they were higher than the survival probabilities of 9 months-old goats; phalanges, are smaller and often more susceptible to taphonomic destruction than the larger and more robust bones, with unfused elements being more likely to suffer more from such taphonomic destruction, potentially artificially increasing the proportion represented by the fused elements and the resulting estimated probability of survival.

Age (in months)	Jarmo, Female	Jarmo, Male	Ali Kosh, Female	Ali Kosh, Male	Ganj Dareh, Female	Ganj Dareh, Male
3	0.99	0.99	0.98	1	0.96	0.98
9	0.98	1	0.95	0.96	0.91	0.94
24	0.96	0.74	0.82	0.54	0.74	0.21
39	0.59	0.46	0.47	0.25	0.27	0.04

Table S3. Human and goat population size estimates. HH: household; it was assumed that there was an average of six people per household; three different levels of goat ownership were used (6, 18 and 36 goats per household).

Site	Area (ha)	People/ha	People	HHs	Goats (6/HH)	Goats (18/HH)	Goats (36/HH)
Jarmo	1.3	100	130	21.7	130	390	780
		300	390	65	390	1170	2340
Ali Kosh	1.43	100	143	23.8	143	429	858
		300	429	71.5	429	1287	2574
Ganj Dareh	0.13	100	13	2.2	13	39	78
		300	39	6.5	39	117	234

Table S4. Partial rank correlation coefficients. The confidence interval is specified in brackets.

	Transitory infectiousness		lifelong infectiousness	
Parameter	R_0=1.5	R_0=2.5	R_0=1.5	R_0=2.5
$\delta_{a=0}$	0.47 (0.4,0.54)	0.33 (0.25,0.41)	0.31 (0.23,0.38)	0.18 (0.09,0.26)
$\delta_{a=1,s=M}$	-0.84 (-0.87,-0.82)	-0.84 (-0.86,-0.81)	-0.73 (-0.76,-0.69)	-0.71 (-0.75,-0.67)
$\delta_{a=1,s=F}$	0.66 (0.61,0.7)	0.5 (0.45,0.57)	0.5 (0.44,0.58)	0.44 (0.38,0.51)
$\delta_{a=2}$	0.98 (0.98,0.99)	0.66 (0.62,0.71)	0.99 (0.99,0.99)	0.99 (0.98,0.99)

Table S5. Partial rank correlation coefficients. The confidence interval is specified in brackets.

Parameter	Transitory infectiousness	Lifelong infectiousness
β	0.87 (0.86,0.87)	0.90 (0.90,0.91)
ASR	-0.49 (-0.52,-0.46)	-0.59 (-0.62,-0.57)
N	0.82 (0.81,0.83)	0.70 (0.68,0.72)

References

1. Zeder M.A. 2008 Animal domestication in the Zagros: an update and directions for future research. In *Archaeozoology of the Near East VIII Travaux de la Maison de l'Orient et de la Méditerranée 49* (eds. Vila E., Gourichon L., Choyke A.M., Buitenhuis H.), pp. 243-277. Lyon, France.
2. Donet J.M., Gunn R.G., Horák F. 1982 Reproduction. In *Sheep and Goat Production* (ed. In Coop I.E.), pp. 57-80. Amsterdam, Netherlands, Elsevier.
3. Gall C. 1996 *Goat breeds of the world*, Technical Centre for Agricultural and Rural Cooperation (CTA).
4. Redding R.W. 1981 Decision making in subsistence herding of sheep and goats in the Middle East, University of Michigan.
5. Weinberg P. 2001 On the status and biology of the wild goat in Daghestan (Russia). *Journal of Mountain Ecology* **6**, 31-40.
6. European Commission Scientific Committee on Animal Health and Animal Welfare. 2001 Brucellosis in sheep and goats. (p. 89.
7. Smith P.E.L. 1976 Reflections on four seasons of excavations at Tapeh Ganj Dareh. In *Proceedings of the Fourth Annual Symposium on Archaeological Research in Iran* (ed. Bagherzadeh F.), pp. 11–22. Tehran, Iranian Centre for Archaeological Research.
8. Hole F., Flannery K.V., Neely J.A. 1969
Prehistory and Human Ecology on the Deh Luran Plain, Memoirs of the Museum of Anthropology 1, The University of Michigan Press, Ann Arbor.
9. Braidwood L., Braidwood R., Howe B., Reed C., Watson P.J. 1983 *Prehistoric Archaeology Along the Zagros Flanks. Studies in Ancient Oriental Civilization 105*. Chicago, University of Chicago Oriental Institute.
10. Köhler-Rollefson I., Rollefson G.O. 1990 The impact of Neolithic subsistence strategies on the environment: the case of Aïn Ghazal, Jordan. Man's Role in the Shaping of the Eastern Mediterranean. In *INQUA/BAI* (pp. 3-14. Rotterdam, AA Balkema.
11. Zorn J.R. 1994 Estimating the population size of ancient settlements: methods, problems, solutions, and a case study. *Bulletin of the American Schools of Oriental Research* **295**, 31-48.
12. Dahl G., Hjort A. 1976 *Having herds: pastoral herd growth and household economy*. Stockholm, Department of Social Anthropology, University of Stockholm.
13. Kramer C. 1982 *Village Ethnoarchaeology*. New York, Academic Press.
